# Automated and Continuous Production of Polymeric Nanoparticles

**DOI:** 10.3389/fbioe.2019.00423

**Published:** 2019-12-17

**Authors:** Giovanni Bovone, Fabian Steiner, Elia A. Guzzi, Mark W. Tibbitt

**Affiliations:** Macromolecular Engineering Laboratory, Department of Mechanical and Process Engineering, ETH Zürich, Zurich, Switzerland

**Keywords:** nanoparticles, drug delivery, flow-based synthesis, automated production, process engineering

## Abstract

Polymeric nanoparticles (NPs) are increasingly used as therapeutics, diagnostics, and building blocks in (bio)materials science. Current barriers to translation are limited control over NP physicochemical properties and robust scale-up of their production. Flow-based devices have emerged for controlled production of polymeric NPs, both for rapid formulation screening (~μg min^−1^) and on-scale production (~mg min^−1^). While flow-based devices have improved NP production compared to traditional batch processes, automated processes are desired for robust NP production at scale. Therefore, we engineered an automated coaxial jet mixer (CJM), which controlled the mixing of an organic stream containing block copolymer and an aqueous stream, for the continuous nanoprecipitation of polymeric NPs. The CJM was operated stably under computer control for up to 24 h and automated control over the flow conditions tuned poly(ethylene glycol)-*block*-polylactide (PEG_5*K*_-*b*-PLA_20*K*_) NP size between ≈56 nm and ≈79 nm. In addition, the automated CJM enabled production of NPs of similar size (*D*_*h*_ ≈ 50 nm) from chemically diverse block copolymers, PEG_5*K*_-*b*-PLA_20*K*_, PEG-*block*-poly(lactide-*co*-glycolide) (PEG_5*K*_-*b*-PLGA_20*K*_), and PEG-*block*-polycaprolactone (PEG_5*K*_-*b*-PCL_20*K*_), by tuning the flow conditions for each block copolymer. Further, the automated CJM was used to produce model nanotherapeutics in a reproducible manner without user intervention. Finally, NPs produced with the automated CJM were used to scale the formation of injectable polymer–nanoparticle (PNP) hydrogels, without modifying the mechanical properties of the PNP gel. In conclusion, the automated CJM enabled stable, tunable, and continuous production of polymeric NPs, which are needed for the scale-up and translation of this important class of biomaterials.

## 1. Introduction

Nanoparticles (NPs) comprise a useful class of biomaterials in modern medicine for the encapsulation and delivery of small molecule drugs, proteins, and nucleic-acid therapies as well as for *in vivo* diagnosis or as agents for improved biomedical imaging (Anselmo and Mitragotri, 2016; Kamaly et al., 2016; Detappe et al., 2017; Shi et al., 2017). NPs are particularly attractive in drug delivery as they can increase the solubility of poorly blood-soluble drugs, enhance drug stability, extend circulation time, and aid transport across biological barriers (Langer, 1998; Tibbitt et al., 2016). Within the field of nanomedicine, aqueous stable polymeric NPs are especially useful as carriers for hydrophobic small molecules, which can be encapsulated directly within the hydrophobic core of the NPs during production without the need for chemical modification of the drug (Cheng et al., 2007; Liu et al., 2010; Bertrand et al., 2017). Drug-loaded NPs can be self-assembled via nanoprecipitation of amphiphilic block copolymers, e.g., poly(ethylene glycol)-*block*-polylactide (PEG_5*K*_-*b*-PLA_20*K*_), PEG-*block*-poly(lactide-*co*-glycolide) (PEG_5*K*_-*b*-PLGA_20*K*_), or PEG-*block*-polycaprolactone (PEG_5*K*_-*b*-PCL_20*K*_). Core-shell NPs have been exploited for systemic delivery of therapeutics following parenteral or oral administration as well as for local delivery following targeted administration in the body (Gref et al., 1994; Song et al., 1997; Westedt et al., 2007; Pridgen et al., 2013, 2015). Beyond the use of core-shell NPs as a stand alone delivery vector, they have recently been exploited as building blocks in the assembly of shear-thinning and self-healing, polymer–nanoparticle (PNP) hydrogels for site specific delivery following local injection (Appel et al., 2015b). PNP hydrogels have also been used as nanocarrier bioinks for additive manufacturing, as a sprayable barrier to prevent tissue adhesion following cardiothoracic surgery, and as a depot for the local release of cytokines and recruitment of immune cells (Fenton et al., 2019; Guzzi et al., 2019; Lopez Hernandez et al., 2019; Stapleton et al., 2019).

Despite the versatility and significant potential of polymeric NPs in biomedicine, translation to the clinic often remains limited by uncontrolled and poorly scalable production (Hickey et al., 2015; Ragelle et al., 2017; Colombo et al., 2018). Clinical application of polymeric NPs, either as a delivery vehicle or as a building block in PNP hydrogels, requires precise control over NP size, efficient drug loading, and scalable production. Polymeric NPs are commonly produced from amphiphilic block copolymers, such as PEG_5*K*_-*b*-PLA_20*K*_, PEG_5*K*_-*b*-PLGA_20*K*_, and PEG_5*K*_-*b*-PCL_20*K*_, by adding a solution of a water-miscible organic solvent, the block copolymer, and, optionally, a hydrophobic drug dropwise to water under vigorous stirring (Fessi et al., 1989; Mora-Huertas et al., 2010). The solvent mixes rapidly with water and the NPs form as the hydrophobic blocks collapse into a kinetically trapped core surrounded by a hydrophilic corona (Nicolai et al., 2010). Conventionally, this nanoprecipitation is carried out in batch with relatively limited throughput as well as minimal control over the production parameters and, thus, NP size or drug loading (Murday et al., 2009). More recently, flow-based devices have been developed for the continuous and tunable production of polymeric NPs via controlled mixing of an organic stream containing the block copolymer and drug with an aqueous stream in micro- or milli-fluidic systems (Johnson and Prud'homme, 2003a; Karnik et al., 2008; Capretto et al., 2012). Precise regulation of the flow rates provides a handle to control NP properties, such as size, by tuning the mixing time (Johnson and Prud'homme, 2003b; Saad and Prud'homme, 2016). Microfluidic devices based on hydrodynamic flow focusing have been used for formulation screening (μg min^−1^), while on-scale production (mg min^−1^ to g min^−1^) was achieved with impinging jet mixers and coaxial jet mixers (CJMs) (Karnik et al., 2008; Lim et al., 2014; Hickey et al., 2015; Liu et al., 2015; Rode García et al., 2018). In our recent work, we developed a CJM from off-the-shelf components for flow-based production of NPs that enabled tunable NP size in both formulation screening mode (~μg min^−1^) and scalable production mode (~mg min^−1^) (Bovone et al., 2019). While flow-based devices have improved the process engineering and production of polymeric NPs, automated processes are needed to offer user-independent scale-up and to minimize human intervention during pharmaceutical production.

In this study, we automated the CJM for continuous, controlled, and scalable production of polymeric NPs. The system exploited computer-controlled syringe pumps to tune the flow rates of the block copolymer solution and aqueous streams within the flow-based device. NPs of specified diameters were formed by tuning the flow rates and the ratio of the two streams and the CJM was operated stably, without human intervention, for up to 24 h. PEG_5*K*_-*b*-PLA_20*K*_ NPs were formed continuously during stable operation, and the size was tuned between ≈56 and ≈79 nm within a single production process. The automated CJM was then used to produce NPs from three distinct polymers, PEG_5*K*_-*b*-PCL_20*K*_, PEG_5*K*_-*b*-PLA_20*K*_, and PEG_5*K*_-*b*-PLGA_20*K*_, with a similar diameter, *D*_*h*_ ≈ 50 nm. In contrast, standard batch nanoprecipitation of PEG_5*K*_-*b*-PCL_20*K*_, PEG_5*K*_-*b*-PLA_20*K*_, and PEG_5*K*_-*b*-PLGA_20*K*_ formed NPs of disparate diameters, *D*_*h*_ ≈ 55, 76, and 60 nm, respectively. Stable operation and tuning of NP size using flow conditions were demonstrated both for dilute (10 mg mL^−1^) and concentrated (50 mg mL^−1^) polymer solutions. In addition, the automated CJM controlled NP size during formulation screening and scale-up of NP production. Model nanotherapeutics were produced with a consistent NP size using the automated CJM and Oil Red O (OR) as a model hydrophobic small molecule drug. Finally, on-scale production of NPs enabled the formation of PNP hydrogels in 0.6 g and 6.0 g batches, without altering the rheological properties of the PNP gels. In total, the automated CJM enabled controlled and scalable production of polymeric NPs with minimal user input, which is essential for the design and translation of nanocarriers and PNP gels for site specific delivery of therapeutics.

## 2. Materials and Methods

### 2.1. Materials

PEG_5k_-*b*-PCL_20k_, PEG_5k_-*b*-PLA_20k_ and PEG_5k_-*b*-PLGA_20k_ were purchased from PolySciTech, a divison of Akina, Inc. (USA). Acetonitrile (ACN) and dimethylformamide (DMF) were purchased from VWR International AG (CH). Ultrapure deionized water (dH_2_O) was freshly filtered using a Milli-Q IQ 7000 from Merck Millipore (CH). All components of the coaxial jet mixer were purchased from BGB Analytik (CH) or Cole-Parmer (US) and are listed in detail in our recent work (Bovone et al., 2019).

### 2.2. Batch Nanoparticle Formation

Block copolymer solutions of 10 mg mL^−1^ or 50 mg mL^−1^ were prepared by dissolving PEG_5k_-*b*-PCL_20k_ or PEG_5k_-*b*-PLA_20k_ in ACN, and PEG_5k_-*b*-PLGA_20k_ in DMF. The organic solution to water ratio, *R*, was defined as

(1) $$\begin{array}{l} R=\frac{V_{organic}}{V_{H_{2}O}} \end{array}$$

For each batch nanoprecipitation, 1 mL of block copolymer solution (*V*_*organic*_) was added drop wise to 10 mL of dH_2_O (*V*_*H*_2_*O*_; *R* = 0.1) under stirring at 650 RPM (Stir bar: 15 mm). All batch nanoprecipitation experiments were performed in triplicate.

### 2.3. Flow-Based Nanoparticle Formation

#### 2.3.1. Experimental Set-Up

The CJM design was based on similar devices used for inorganic particle synthesis and a recently developed device from our group for the flow-based production of polymeric nanoparticles (Baber et al., 2015; Bovone et al., 2019). The CJM was assembled from off-the-shelf components within minutes. In brief, an inner fused silica capillary was centered coaxially to an outer PTFE tube (ID = 1/32"; OD = 1/16"; L = 12 cm). Two different fused silica capillaries were used depending on the selected *R* for NP production; for *R* = 0.005 the capillary dimensions were OD = 363 μm and ID = 100 μm, and for *R* = 0.1 the capillary dimensions were OD = 363 μm and ID = 150 μm. All components were assembled in a PEEK T-junction. The alignment of the capillary and the main channel was the most difficult step and extra care should be taken here to ensure proper alignment of the device. The effect of alignment on NP production was tested previously by disassembling and reassembling the device after each synthesis (Bovone et al., 2019). NP fabrication with different capillary alignments showed a variability of up to ±10 nm. As this issue was studied extensively in our previous work, each automated production experiment was conducted using the same device and the inner capillary was exchanged as needed. The CJM was designed such that the block copolymer solution flowed through the inner fused silica capillary and the dH_2_O flowed through the outer PTFE channel. The fluid streams were delivered from 2.5, 10, or 50 mL gas-tight syringes (SETonic) operated by computer-controlled syringe pumps (CETONI NeMESYS Low Pressure 29:1 gear & CETONI NeMESYS Low Pressure 14:1 gear). The pumps, and thus the flow rates of the fluid streams, were controlled externally by a LabView (National Instruments, USA) script provided in the Supplementary Material (**Section S1.2**), which utilized functions from the Qmix software developement kit (CETONI).

#### 2.3.2. CJM NP Formation

For nanoprecipitation in the CJM, the block copolymers PEG_5k_-*b*-PCL_20k_, PEG_5k_-*b*-PLA_20k_, or PEG_5k_-*b*-PLGA_20k_ were first dissolved in ACN or DMF at concentrations of 10 mg mL^−1^ for dilute NP formulation screening or 50 mg mL^−1^ for concentrated NP production. DMF was used for block copolymers that nanoprecipitate into larger NPs, i.e., PEG_5k_-*b*-PLGA_20k_, as NPs produced with DMF were smaller than those produced with ACN, in preliminary experiments. In CJM experiments, the organic solution to dH_2_O ratio, *R*, was defined as:

(2) $$\begin{array}{l} R=\frac{Q_{organic}}{Q_{H_{2}O}} \end{array}$$

where *Q*_*organic*_ and *Q*_*H*_2_*O*_ represent the volumetric flow rates of the respective fluid streams. NP formation in dilute conditions was performed at *R* = 0.005. Concentrated NP production was performed at *R* = 0.1. Volumetric flow rates of dH_2_O used in our study ranged from ~1 to ~35 mL min^−1^, whereas the organic solution volumetric flow rate ranged from ~50 μL min^−1^ to ~4 mL min^−1^. The Reynolds number, *Re*, for each experiment was calculated by estimating the viscosity and density of the final solvent-water mixture from literature values (Aminabhavi and Gopalakrishna, 1995). For the *Re* calculations, the inner diameter of the water PTFE tube was used as the characteristic length. The velocity was calculated based on the inner cross-sectional area of the outer tube of the CJM. The experiments were performed in cycles, which were determined by the complete refill and dispensing of the syringes.

#### 2.3.3. Automated NP Production

**Figure 1:**
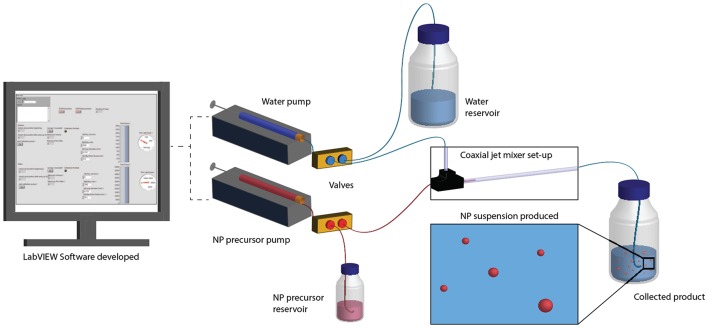
NP production in the automated CJM. The set-up was designed for the production of NPs and included syringe pumps, valves, and reservoirs that were computer-controlled. A LabView program and a GUI enabled automation of the CJM to operate independently during NP production.

To automate NP production, the CJM was connected to computer-controlled syringe pumps that dispensed the block copolymer solution, optionally containing a model drug, and dH_2_O (Figure 1). A graphical user interface (GUI) was designed in LabView to control syringe filling from reservoirs of the two solutions and dispensing through the CJM into a collection reservoir. This enabled NP production without user intervention outside of system set-up, sample collection, and formulation switching. The flow rates or Reynolds number, *Re*, as well as the ratio between the volumetric flow rates of the organic and aqueous streams, *R*, were varied to control NP size.

For process automation, the NP production process was divided into multiple operational steps, which were individually programmed in LabView. During the first steps, the set-up was paired to the LabView program, initialized, and all syringes and valves were mounted. The production cycle was initiated with the refill of the syringes with water and polymer solution. Prior to starting the NP production, the flow rates were gradually increased and both the water and NP precursor solution were collected back into the respective reservoirs. When the flow rates stabilized, the valves redirected the flow into the main channel for NP production until one of the syringes was emptied to 15% of its total volume. The valves switched the flow back to the reservoirs, the flow rates were gradually decreased, and the cycle started over with refilling both syringes. In some experiments, the polymer or formulation solution was changed in between cycles. In this case, the CJM was equilibrated over 3 cycles of washing to accommodate the new formulation solution. A detailed description of all steps and a summary of all data is provided in the Supplementary Material (**Sections S1.1** and **S2**).

### 2.4. NP Characterization

The hydrodynamic diameter, *D*_*h*_, of the synthesized NPs was characterized via dynamic light scattering (DLS) on a ZetaSizer Nano ZS (Malvern, UK). A NP suspension volume of ~1 mL was measured at a scattering angle of 173° at 25°C. NP suspensions formed at *R* = 0.1 were diluted in dH_2_O by a factor of 10. There was no change in observed *D*_*h*_ upon dilution (Bovone et al., 2019). NPs produced at *R* = 0.005 were analyzed as collected. The z-average hydrodynamic diameter, *D*_*h*_, and the dispersity, Ð, were calculated over three measurements per sample. The dispersity was calculated according to ISO 22412:2017 (2017-02):

(3) $$\begin{array}{l} \text{Ð}=\frac{\sigma^{2}}{2\bar{\Gamma }} \end{array}$$

where $\bar{\Gamma }$ represents the scattered light intensity-weighted average and σ represents the standard deviation of the distribution function.

### 2.5. Synthesis and Characterization of Drug Delivery Systems

#### 2.5.1. Encapsulation of Small Molecules

A solution of OR (0.5 mg mL^−1^) and PEG_5*K*_-*b*-PLA_20*K*_ (50 mg mL^−1^) in ACN was prepared to achieve a theoretical drug loading of 1%.

(4) $$\begin{array}{l} \text{Theoretical drug loading}=\text{tDL}=\frac{m_{\text{drug in formulation}}}{m_{\text{total formulation}}}\cdot 100\% \end{array}$$

where *m*_drug in formulation_ represents the total mass of model therapueutic used in the formulation and *m*_total formulation_ is calculated by the sum of the block copolymer mass and of the model therapeutic mass. The formulation solution and dH_2_O were injected into the CJM at ~3.2 mL min^−1^ and ~32 mL min^−1^, respectively (*R* = 0.1, *Re* = 1016). Approximately three samples of 10 mL of the produced NPs were collected during each cycle. After NP production, the hydrodynamic diameter of each sample was measured via DLS. OR was quantified via UV-Vis spectroscopy at λ = 520 nm according to the protocol explained in the Supplementary Material (**Section S1.3**). The effective OR loading into the NPs was defined as

(5) $$\begin{array}{l} \text{Effective drug loading}=\text{eDL}=\frac{m_{\text{drug after filtration}}}{m_{\text{total formulation after filtration}}}\cdot 100\% \end{array}$$

where *m*_drug after filtration_ and *m*_total formulation after filtration_ represent the respective residual mass of drug and of total matter after NP work-up.

#### 2.5.2. Synthesis of Polymer–Nanoparticle Hydrogels

50 mg mL^−1^ PEG_5*K*_-*b*-PLA_20*K*_ NPs were synthesized in the automated CJM with *Q*_*dH*_2_*O*_ ~32 mL min^−1^ and *Q*_*organic*_ ~3.2 mL min^−1^ (*R* = 0.1, *Re* = 1016). For the assembly of 0.6 g PNP hydrogels, ~15 mL of the NP suspension were utilized, and the remaining ~150 mL of the NP suspension were used for scaling up the PNP hydrogels to 6 g. The PNP hydrogels were composed by 2 %w/w hydroxypropylmethylcellulose (HPMC) and 15 %w/w PEG_5*K*_-*b*-PLA_20*K*_ NPs (HPMC:NP, 2:15 %w/w). Further details on the synthesis of PNP hydrogels were reported in the Supplementary Material (**Section S1.4**).

#### 2.5.3. Rheological Characterization of Polymer–Nanoparticle Hydrogels

Rheological tests were performed using a strain-controlled shear rheometer (MCR 502; Anton Paar; CH) fitted with a Peltier stage (*T* = 37°C). During the measurements, silicon oil was used to prevent evaporation. All experiments were performed using a 25 mm cone-plate geometry with a 2° truncation angle. The storage modulus, *G*′, loss modulus, *G*″, and the loss factor, $\tan\left(\delta \right)=\frac{G^{\prime \prime }}{G^{\prime }}$, were measured with an oscillatory strain amplitude sweep (γ = 0.1–1000%) at a constant angular frequency (ω = 10 rad s^−1^). Oscillatory step strain recovery experiments were performed at ω = 10 rad s^−1^ to investigate the cyclic recovery from a high strain interval (1000%, 4 min) followed by a low strain interval (0.3%, 8 min). The shear-thinning properties of PNP hydrogels were investigated with rotational shear rate ramp tests ($\frac{\delta \gamma }{\delta t}$ = 0.1–100 s^−1^).

## 3. Results and Discussion

### 3.1. Automated CJM for NP Production

A coaxial jet mixer (CJM) was assembled based on previous designs (Baber et al., 2015; Bovone et al., 2019) and automated for flow-based nanoprecipitation of polymeric NPs. The resulting NPs were compared to those produced by standard batch nanoprecipitation. A solution of PEG_5*K*_-*b*-PLA_20*K*_ in ACN (50 mg mL^−1^) was nanoprecipitated in dH_2_O under flow (*R* = 0.1, *Re* = 1016). In the CJM, the PEG_5*K*_-*b*-PLA_20*K*_ NPs formed with *D*_*h*_ ≈ 78 nm (Figure S7A). The same polymer formed NPs with *D*_*h*_ ≈ 94 nm via batch nanopreciptiation (*R* = 0.1). In both cases, the NPs formed with Ð < 0.1, indicating a narrow size distribution and effective NP formation. Thus, the CJM produced PEG_5*K*_-*b*-PLA_20*K*_ NPs of similar size and quality to standard batch nanoprecipitation.

As the CJM was programmed with unit operations for automatic syringe filling and dispensing, the system could operate independent of an operator following set-up and filling of the organic and aqueous reservoirs. This enabled the CJM to produce NPs continuously with standard lab-scale syringes (up to 50 mL volume) over extended periods of time. For example, the respective reservoirs were filled with ~830 mL of PEG_5*K*_-*b*-PLA_20*K*_ in ACN (10 mg mL^−1^) and 8.3 L of dH_2_O and the automated CJM was programmed to produce NPs under flow (*R* = 0.1, *Re* = 1016) for 24 h without human intervention. The automated CJM operated stably without leaks or clogging over the 24 h experiment. The ability to operate continuously over extended periods of time is a major advantage of the automated CJM and is essential for scalable production of NPs.

### 3.2. Stable Operation of the CJM and Tuning of NP Size During Operation

**Figure 2:**
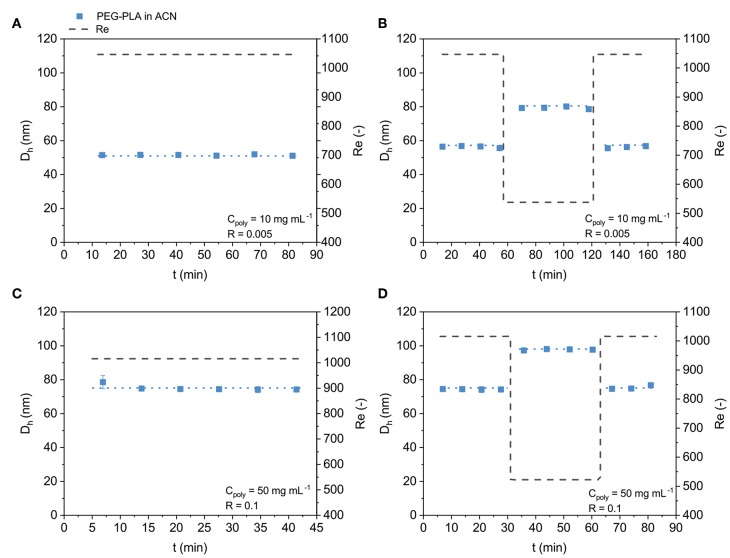
Stable PEG_5*K*_-*b*-PLA_20*K*_ NP production and size control in the automated CJM. **(A)** PEG_5*K*_-*b*-PLA_20*K*_ was nanoprecipitated over several automated cycles (*Re* = 1047) showing reproducible and stable NP size over the whole production process. **(B)** Change in *Re* between 1,047 (*D*_*h*_ ≈ 56 nm) and 538 (*D*_*h*_ ≈ 79 nm) demonstrated control over NP size by altering flow conditions. **(C)** Concentrated automated production of NPs was carried out with commonly used block copolymers at 50 mg mL^−1^ and R = 0.1. PEG_5*K*_-*b*-PLA_20*K*_ NP production remained stable (*Re* = 1016), *D*_*h*_ ≈ 75 nm, with a production rate of ~40 g day^−1^. **(D)** Re controlled NP size for the concentrated PEG_5*K*_-*b*-PLA_20*K*_ formulations where *Re* = 1016 lead to *D*_*h*_ ≈ 74 nm and *Re* = 522 lead to *D*_*h*_ ≈ 98 nm.

In an initial test, we demonstrated that the automated CJM could operate without leaks or clogging for up to 24 h. Here, we investigated the stability of NP production over time and dynamic tuning of NP size during operation. First, NPs were produced from PEG_5*K*_-*b*-PLA_20*K*_ in ACN (10 mg mL^−1^). The CJM was operated (*Re* = 1047, *R* = 0.005) for 12 filling and dispensing cycles, equivalent to 80 min of operation time. Three discrete samples were collected directly from the exit stream of the CJM to monitor NP properties every second production cycle (Figure 2A). An additional sample was taken from the NP suspension collection reservoir after the 80 min of operation. During the course of continuous NP production, NP size remained stable with *D*_*h*_ ≈ 51 nm and Ð = 0.06 − 0.09 for the discrete samples. The diameters from the discrete samples were consistent with the NP diameter of the samples from the collection reservoir. Thus, NP size and quality remained constant during continuous operation, highlighting the ability to produce NPs stably with the automated CJM, with dilute conditions.

Another useful feature of the automated CJM is the ability to modify the flow rates of the two fluid streams independently to control *Re* and *R* and, therefore, NP size (Bovone et al., 2019). Here, NPs were produced from PEG_5*K*_-*b*-PLA_20*K*_ in ACN (10 mg mL^−1^) with *D*_*h*_ ≈ 56 nm at *Re* = 1047 over eight filling and dispensing cycles (Figure 2B). The size was then changed to *D*_*h*_ ≈ 79 nm (*Re* = 538) for the next eight cycles and then back to 56 nm (*Re* = 1047) for an additional six cycles. These results demonstrated that NP size could be tuned dynamically during continuous operation by altering the flow conditions, such as *Re*.

### 3.3. Scalable and Automated NP Production

Beyond continuous and controlled NP prodution, scale-up remains a major hurdle to clinical translation of nanotherapeutics and PNP hydrogels for site specific delivery (Liu et al., 2015, 2018). To increase the NP production rate in the automated CJM, the block copolymer concentration was increased to 50 mg mL^−1^ and *R* was increased to 0.1. Here, NPs were produced from PEG_5*K*_-*b*-PLA_20*K*_ in ACN under flow with the concentrated block copolymer solution. The CJM was operated (*Re* = 1016) for 6 filling and dispensing cycles, equivalent to 40 min of operation time. Discrete samples were collected directly from the exit stream of the CJM to monitor NP properties every cycle (Figure 2C). An additional sample was taken from the NP suspension collection reservoir after the 40 min of operation. During the course of continuous NP production, the NP size remained stable with *D*_*h*_ ≈ 75 nm and Ð = 0.07 − 0.08 for the discrete samples. The *D*_*h*_ and Ðof the sample from the collection reservoir were 75 nm and 0.07, respectively. Thus, NP size and quality remained constant during continuous operation also in production mode. The increased size relative to the dilute condition was expected as C_poly_ and *R* are both known to influence NP size (Karnik et al., 2008; Bovone et al., 2019). With the current setup, a production rate of ~40 g day^−1^ was possible. This calculation accounted for the downtime needed for the refilling steps, which was the most time consuming step in this design of the CJM. This is a significant improvement over the standard batch nanoprecipitation production rate. Further, a theoretical production rate of 230 g day^−1^ could be achieved with the automated CJM given additional syringes and pumps, such that some pumps could be refilling while others are dispensing. The CJM was tested for automated *D*_*h*_ tuning during concentrated NP production. NPs were produced at *Re* = 1016 for 4 cycles, followed by 4 cycles at *Re* = 522, and returning to *Re* = 1016 for a final 3 cycles (Figure 2D). These conditions produced PEG_5*K*_-*b*-PLA_20*K*_ NPs of *D*_*h*_ ≈ 74, 98, and 75 nm, respectively. The data demonstrates that the CJM retained the ability to tune NP size also during concentrated NP production.

### 3.4. Decoupling NP Formulation From Size

**Figure 3:**
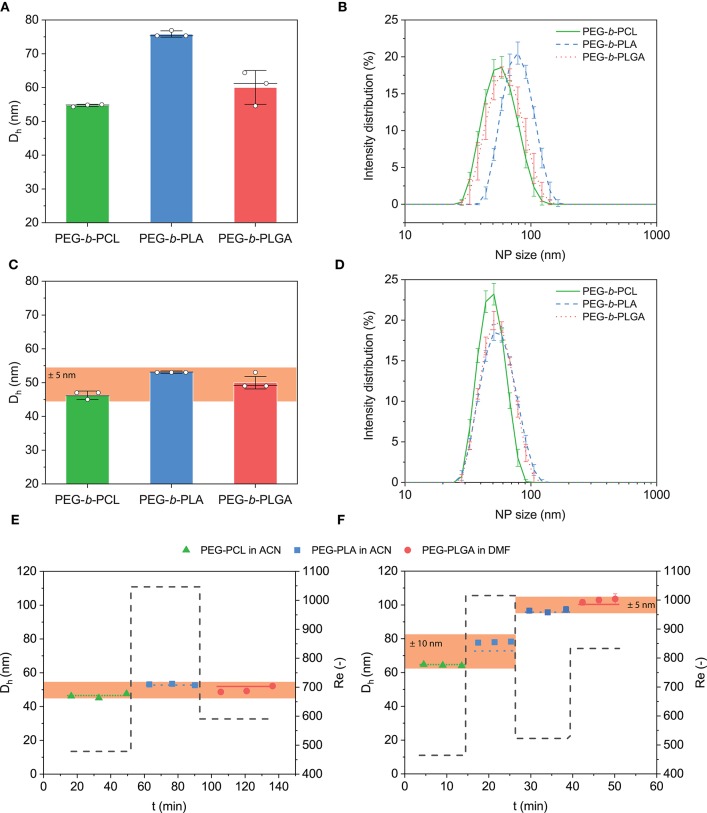
Nanoprecipitation of common block copolymers via batch and with the automated CJM. **(A)** Diluted screening of NP size was carried out with commonly used block copolymers at concentrations of 10 mg mL^−1^ and *R* = 0.005. Batch nanoprecipitation (10 mg mL^−1^, *R* = 0.005) of PEG_5*K*_-*b*-PCL_20*K*_ or PEG_5*K*_-*b*-PLA_20*K*_ dissolved in ACN and PEG_5*K*_-*b*-PLGA_20*K*_ dissolved in DMF produced NPs of *D*_*h*_ ≈ 76, 55, and 60 nm, respectively with Ð < 0.1. **(B)** Corresponding size distribution of NPs produced in batch. **(C)** Flow control in the CJM enabled production of NPs with uniform size from chemically distinct formulations, *D*_*h*_ = 50±5 nm. NP production was carried out at *Re* = 478, 1047, and 591, respectively for PEG_5*K*_-*b*-PCL_20*K*_, PEG_5*K*_-*b*-PLA_20*K*_, and PEG_5*K*_-*b*-PLGA_20*K*_. **(D)** Relative size distribution of the CJM produced NPs. **(E)** The automated flow-controlled CJM enabled the continuous production of NPs with similar size *D*_*h*_ ≈ 50 nm from the chemically distinct block copolymers over multiple cycles. **(F)** Concentrated automated production of NPs was carried out with commonly used block copolymers at 50 mg mL^−1^ and R = 0.1. Pairs of similar diameter but chemically distinct NPs were produced in the CJM. NPs were produced with *D*_*h*_ ≈ 65 − 78 nm from PEG_5*K*_-*b*-PCL_20*K*_ (*Re* = 478) and PEG_5*K*_-*b*-PLA_20*K*_ (*Re* = 1016). Subsequently, NPs were produced with *D*_*h*_ ≈ 96 − 103 nm from PEG_5*K*_-*b*-PLA_20*K*_ NPs (*Re* = 522) and PEG_5*K*_-*b*-PLGA_20*K*_ (*Re* = 833).

To further demonstrate the utility of flow control over NP size, the CJM was exploited to prepare NPs from three common block copolymers used for drug delivery purposes, namely PEG_5*K*_-*b*-PLA_20*K*_, PEG_5*K*_-*b*-PCL_20*K*_, and PEG_5*K*_-*b*-PLGA_20*K*_. First, PEG_5*K*_-*b*-PLA_20*K*_ and PEG_5*K*_-*b*-PCL_20*K*_ were each dissolved in ACN and PEG_5*K*_-*b*-PLGA_20*K*_ was dissolved in DMF. NPs were prepared via dilute batch nanoprecipitation (10 mg mL^−1^ block copolymer solution, *R* = 0.005) with *D*_*h*_ = 55 ± 1, 76 ± 1, and 60 ± 5 nm, for PEG_5*K*_-*b*-PCL_20*K*_, PEG_5*K*_-*b*-PLA_20*K*_, and PEG_5*K*_-*b*-PLGA_20*K*_ respectively (Figure 3A). All NPs formed with low dispersity (Ð < 0.1) and unimodal size distributions (Figure 3B). The results show that batch nanoprecipitation was able to produce NPs with low dispersity in a simple manner; however, formulations using different block copolymer chemistries resulted in NPs of distinct sizes. To test the ability of the CJM to decouple NP size from block copolymer chemistry, the device was used to produce NPs from each block copolymer with a similar size, *D*_*h*_ ≈ 50 nm, by tuning the flow conditions (Figure 3C). This was achieved with *Re* = 478, 1047, and 591 for PEG_5*K*_-*b*-PCL_20*K*_, PEG_5*K*_-*b*-PLA_20*K*_, and PEG_5*K*_-*b*-PLGA_20*K*_, respectively. The resulting NP populations were similar both in their hydrodynamic diameter and in their size distribution (Figure 3D). This demonstrated that automated control of the flow conditions in the CJM was sufficient to produce NPs with similar size and dispersity from chemically distinct block copolymers, which formed NP of different size in batch nanoprecipitation. That is, the automated CJM was able to decouple *D*_*h*_ from the chemical composition of the NP.

Further, the CJM was tested on the stability of NP production with PEG_5*K*_-*b*-PCL_20*K*_, PEG_5*K*_-*b*-PLA_20*K*_, and PEG_5*K*_-*b*-PLGA_20*K*_ over time. NPs with *D*_*h*_ ~ 50 nm were produced from PEG_5*K*_-*b*-PCL_20*K*_ in ACN (10 mg mL^−1^) for six cycles (*Re* = 478) (Figure 3E). Then, NPs of a similar diameter were produced from PEG_5*K*_-*b*-PLA_20*K*_ in ACN (10 mg mL^−1^, *Re* = 1,047) and PEG_5*K*_-*b*-PLGA_20*K*_ in DMF (10 mg mL^−1^, *Re* = 591) for an additional six cycles each. The automated CJM was also tested on the concentrated production of NPs using the same block copolymers. In concentrated batch nanoprecipitation, these polymers (50 mg mL^−1^, *R* = 0.1) formed NPs with *D*_*h*_ ≈ 65, 94, and 127 nm, respectively (Figure S7B). Here, pairs of similar diameter but chemically diverse NPs were produced (Figure 3F). In the first 3 cycles, PEG_5*K*_-*b*-PCL_20*K*_ was nanoprecipitated at *Re* = 464 and PEG_5*K*_-*b*-PLA_20*K*_ at *Re* = 1016, producing NPs in the range of *D*_*h*_ ≈ 65 − 78 nm. In the subsequent 3 cycles, the size of PEG_5*K*_-*b*-PLA_20*K*_ NPs was increased (*Re* = 522) and matched to the one of PEG_5*K*_-*b*-PLGA_20*K*_ (*Re* = 833), forming NPs of *D*_*h*_ ≈ 96 − 103 nm. These results confirmed that stable NP size control can be achieved in the automated CJM also for concentrated formulations, independent of the chemistry of the block copolymer. The CJM device decoupled NP size from the specific formulation, enabling the tuning of NP dimensions as a separate design parameter of polymeric NPs. These data further demonstrated the ability of the automated CJM to produce particles continuously and stably both in formulation screening and production modes.

### 3.5. Formation of Drug-Loaded NPs

**Figure 4:**
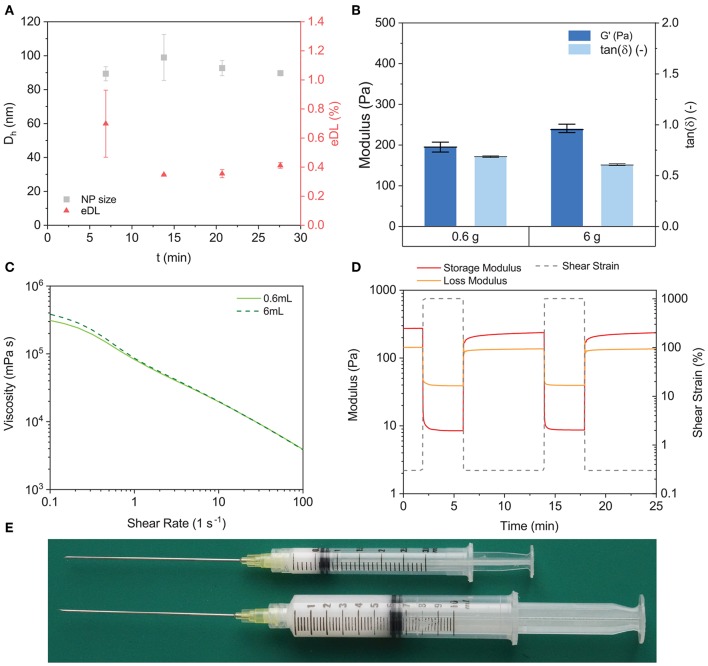
Synthesis of nanotherapeutics and scale-up of PNP hydrogels. **(A)** PEG_5*K*_-*b*-PLA_20*K*_ (50 mg mL^−1^) and OR (0.5 mg mL^−1^, *tDL* ≈ 1%) were nanoprecipitated at *Re* = 1016. NP size was stable over the production *D*_*h*_ ≈ 93 nm and the eDL converged to ~0.4%. **(B)** CJM NP synthesis allowed the production of PNP hydrogels (HPMC:NP, 2:15 wt%) on the 0.6 g and on the 6.0 g scale. Dynamic moduli were measured via oscillatory strain amplitude sweeps (γ = 0.1–1000%, ω = 10 rad s^−1^) **(C)** Rotational shear rate ramp ($\frac{d\gamma }{dt}$ = 0.1–100 s^−1^) of both the 0.6 g and of the 6.0 g PNP hydrogels showed decrease in viscosity with increasing shear rate demonstrating that the shear-thinning properties were retained at both scales. **(D)** The self-healing behavior of the 6.0 g PNP hydrogel was characterized with step strain measurements by alternating intervals of high (1000%, ω = 10 rad s^−1^) and low (0.3%, ω = 10 rad s^−1^) shear strain amplitude. The scaled-up PNP hydrogel demonstrated its ability to self-heal, as reported in literature and similarly to the 0.6 g hydrogel (Figure S8). **(E)** 0.6 g and 6.0 g PNP hydrogels were produced and loaded in plastic syringes.

One of the main applications of polymeric NPs is for the formation of drug-loaded nanotherapeutics. Here, we tested the ability of automated CJM to produce drug-loaded NPs in a stable manner. OR was selected as a model drug owing to its hydrophobicity and ease of detection. A solution of PEG_5*K*_-*b*-PLA_20*K*_ (50 mg mL^−1^) and OR (0.5 mg mL^−1^) in ACN was used as the organic stream and NPs were nanoprecipitated from dH_2_O under flow (*Re* = 1016, *R* = 0.1) in the automated CJM with a target OR loading of 1%. OR-loaded NPs were produced stably over four cycles, or 30 min of operation time, with *D*_*h*_ ≈ 89 − 99 nm (Figure 4A). The OR loading in the first cycle was ~0.7 ± 0.2% and ~0.4 ± 0.1% for each of the subsequent cycles. The reason for the discrepancy between the first and the subsequent cycles was not clear and we hypothesized that it was caused by a transient effect during the first phase of CJM operation. These results demonstrated that the automated CJM was also useful for the stable production of model nanotherapeutics.

### 3.6. Fabrication of Polymer–Nanoparticle (PNP) Hydrogels

An emerging application of polymeric NPs is as building blocks for the assembly of PNP hydrogels (Appel et al., 2015a,b; Guzzi et al., 2019; Lopez Hernandez et al., 2019; Stapleton et al., 2019; Steele et al., 2019). PNP hydrogels form spontaneously upon simple mixing of an appropriately paired polymer, e.g., hydroxypropylmethylcellulose (HPMC) or C12-functionalized hyaluonic acid, and a concentrated solution of core-shell NPs under aqueous conditions. PNP hydrogels are shear-thinning and self-healing owing to the reversible interactions between the polymers and NPs, and have been used for site specific delivery of therapeutics following injection *in vivo* (Appel et al., 2015b; Fenton et al., 2019; Steele et al., 2019). The clinical potential of these materials is significant; however, biomedical PNP gels are currently limited in the scale of their production. As PNP gels form via admixing of a polymer solution and a NP solution, the main limitation to scale is the availability of large amounts of high quality polymeric NPs (Yu et al., 2016). Therefore, we leveraged the automated CJM to produce ~1 g of PEG_5*K*_-*b*-PLA_20*K*_ NPs (*D*_*h*_ ≈ 80 nm). The NPs were concentrated using centrifugal filter units (Amicon Ultra-15, Ultracel membrane, MWCO ≈ 50 kDa; Millipore) to a stock concentration of 20%w/w in dH_2_O. From this suspension, two PNP gel samples were prepared at a final concentration of 2%w/w HPMC and 15%w/w NPs at a standard production of 0.6 g and a scaled production of 6.0 g. The rheological properties, *G*′ ≈ 220 Pa and a tan(δ) ≈ 0.65, were consistent for the two scales (Figure 4B). The scaled version of the PNP gel maintained a high degree of shear-thinning (Figure 4C) and rapid self-healing (Figure 4D and Figure S8). This demonstrated that the automated CJM enabled more efficient and higher scale production of injectable PNP gels (Figure 4E), which could be useful for site specific delivery of therapeutics following local injection.

## 4. Conclusion

In this work, we engineered an automated CJM operated by computer-controlled syringe pumps and valves. A LabView program and a GUI were designed to enable external control over the cycles of refilling, dispensing, and washing and, therefore, NP production. The automated CJM was operated for up to 24 h without user intervention and enabled robust and stable production of PEG_5*K*_-*b*-PLA_20*K*_ NPs. PEG_5*K*_-*b*-PLA_20*K*_ NP diameter was tuned by controlling the flow conditions, *D*_*h*_ ≈ 56 or 79 nm at *Re* = 1047 or 538, respectively. Flow-control in the automated CJM enabled nanoprecipitation of chemically diverse block copolymers, PEG_5*K*_-*b*-PCL_20*K*_, PEG_5*K*_-*b*-PLA_20*K*_, and PEG_5*K*_-*b*-PLGA_20*K*_, with similar size, *D*_*h*_ ≈ 50 nm. Stable, robust, and controlled production of NPs was demonstrated both for dilute (10 mg mL^−1^, *R* = 0.005, production rate ~ 0.3 mg min^−1^ including refill time) as well as for concentrated NP formulations (50 mg mL^−1^, *R* = 0.1, production rate ~ 30 mg min^−1^ including refill time). A key application of the automated CJM would be for the production of nanotherapeutics, therefore, a model small molecule drug, OR, was encapsulated in PEG_5*K*_-*b*-PLA_20*K*_ NPs. NPs of similar size, *D*_*h*_ ≈ 93 nm, and effective OR loading, eDL ~ 0.4%, were produced stably over several cycles with a production rate of ~ 30 mg min^−1^. NPs are not only attractive for systemic drug delivery, but also as a structural component for the formation of injectable PNP hydrogels for site specific drug release. NPs produced with the automated CJM were used for scale-up of PNP hydrogel formation from 0.6 g to 6.0 g. The mechanical properties of the PNP hydrogels were invariant of scale. Thus, the engineered CJM enabled automated, controlled, and continuous synthesis of various common polymeric NPs at different production rates, and for the synthesis of both systemic and local drug delivery systems. Further developments of these fluidic platforms could be instrumental for future translation of nanomaterials to production scales.

## Data Availability Statement

The datasets for this study are available upon request.

## Author Contributions

GB and MT conceived of the project and designed the experiments. GB, FS, and EG performed the experiments. FS developed the program to control the automated CJM. GB, FS, EG, and MT analyzed the data and prepared the figures. GB, FS, and MT wrote the manuscript.

### Conflict of Interest

The authors declare that the research was conducted in the absence of any commercial or financial relationships that could be construed as a potential conflict of interest.
