# Supplementary material for: Automated and Continuous Production of Polymeric Nanoparticles
Source: Front Bioeng Biotechnol. 2019 Dec 17;7:423. doi: 10.3389/fbioe.2019.00423 (PMC6927919; doi:10.3389/fbioe.2019.00423)
Supplement: Supplementary file 1 [file Data_Sheet_1.pdf]

Supplementary Material

Automated and continuous production of polymeric nanoparticles

Giovanni Bovone[1], **Fabian Steiner**[1], **Elia A. Guzzi**[1], **Mark W. Tibbitt**[1]

[1] Macromolecular Engineering Laboratory, Department of Mechanical and Process Engineering, ETH Zürich, 8092 Zürich, Switzerland

1 SUPPLEMENTARY METHODS

1.1 Description of the automated NP production

The automated CJM was monitored with an inverted microscope (Figure S1). The set-up included two syringe pumps equipped with two-way valves, three reservoirs (water, NP formulation solution, and produced NP suspension), as well as a PC for automated control of the process. The CJM automation

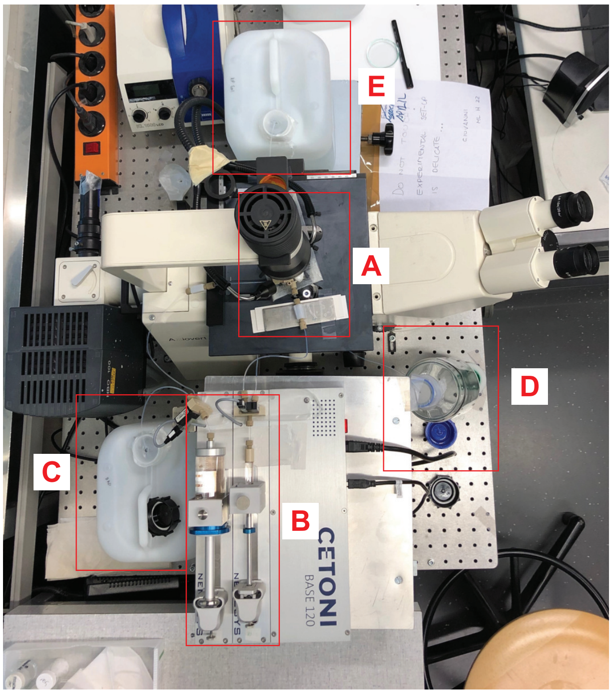

Figure S1. Picture of the automated CJM setup. The CJM was placed on the inverted microscope (A) which was connected to two syringes mounted on the corresponding pumps (B). Two-way valves directed the flow from the reservoirs into the syringes and vice versa from the syringes into the CJM. The solutions for NP production were loaded in a water reservoir (C) and in a formulation solution reservoir (D). The formed NPs were collected in a NP suspension container (E). A PC was utilized for process control (not shown).

was performed by controlling syringe pumps and set-up valves with the software LabView and Qmix Elements. The developed system allowed precise tuning of the flow rates and of the composition. The flow was directed to the CJM or to the respective reservoirs. The following parameters had to be input in the provided user interface (Figure S2):

- Syringe piston strokes
- Syringe diameters
- Refill flow rates
- Production flow rates

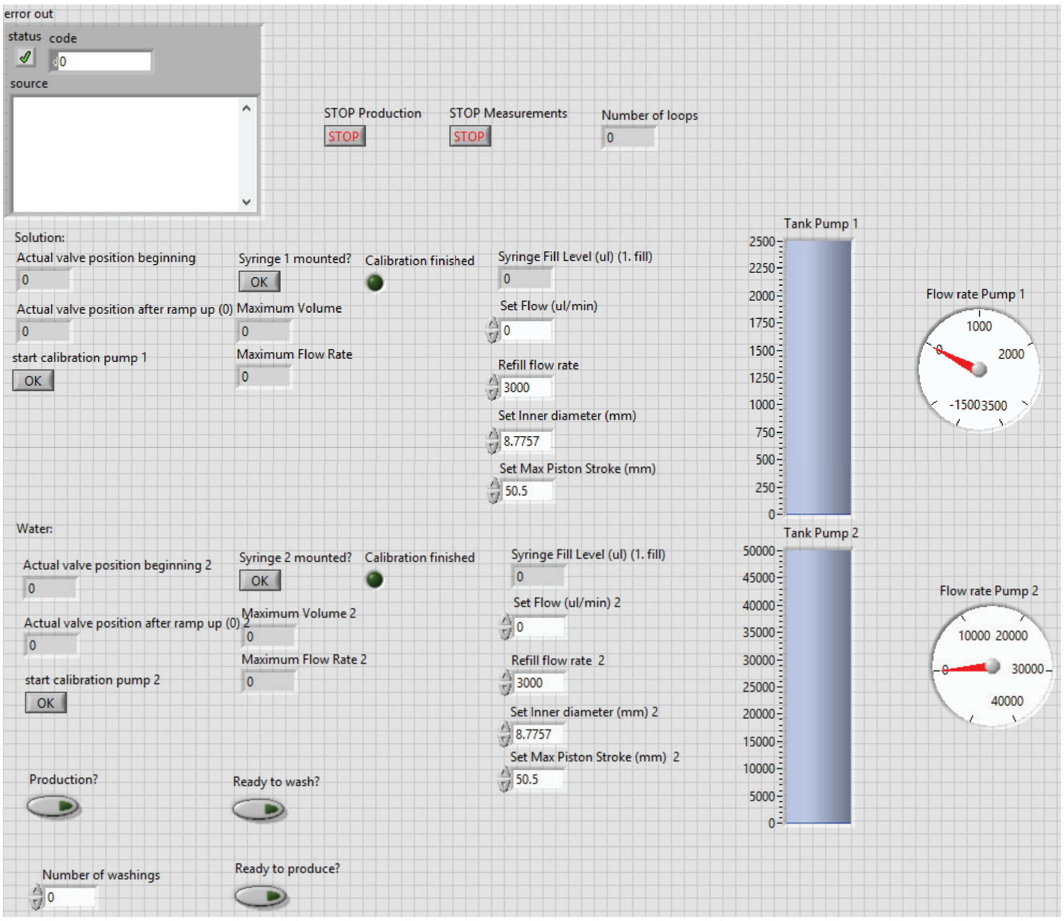

Figure S2. A graphic user interface was designed to control the syringe pumps in a user-friendly manner.

The first production steps were devoted to set-up initialization (Figure S3).

- During *system initialization step* the syringe pumps were connected to the computer. The pump and valve configuration were loaded and the syringe parameters (piston stroke, L_{piston}, and diameter, D_{inner}) were input.
- In the *reference move and syringe mounting steps* the pump pistons were moved to the starting position. Subsequently, the syringes were mounted.
- During *process parameters determination step* the syringe flow parameters were input and the software calculated maximum allowed volumes and flow rates.
- During the *first syringe refill step* the syringes were refilled with water and NP precursor solutions from the respective reservoirs.

During each NP production cycle, the following set of operational steps were executed automatically and repeated for the desired number of cycles. During the refill, ramp up, and ramp down steps the flow was directed from the syringes to the respective reservoirs. During the NP production step, the valves directed the flow into the CJM .

- The flow parameters were read by the program at the beginning of each cycle. This allowed to tune or update the flow rates between each production cycle. During the *gradual flow increase step* the flow rates were linearly increased to the set point over a period of 10 s.

- When the desired flow rate was reached, NP production was started by redirecting the flow into the CJM and initiating nanoprecipitation.
- The production of NPs continued over the *NP production step* and was terminated when less than 15% of the maximum volume remained in either syringe. This ensured that enough solution volume remained to gradually decrease the flow rates without any abrupt cessation of flow.
- NP production was terminated by switching the valves back to the respective reservoirs (*switch valves step*).
- The *gradual flow decrease step* allowed a linear decrease of the flow rates over 10 s until the flow was stopped.
- The aim of the *refill step* was to prepare the syringes for the subsequent NP production run. As the refill flow rates were lower than the production flow rates, this step was most time consuming during each cycle.

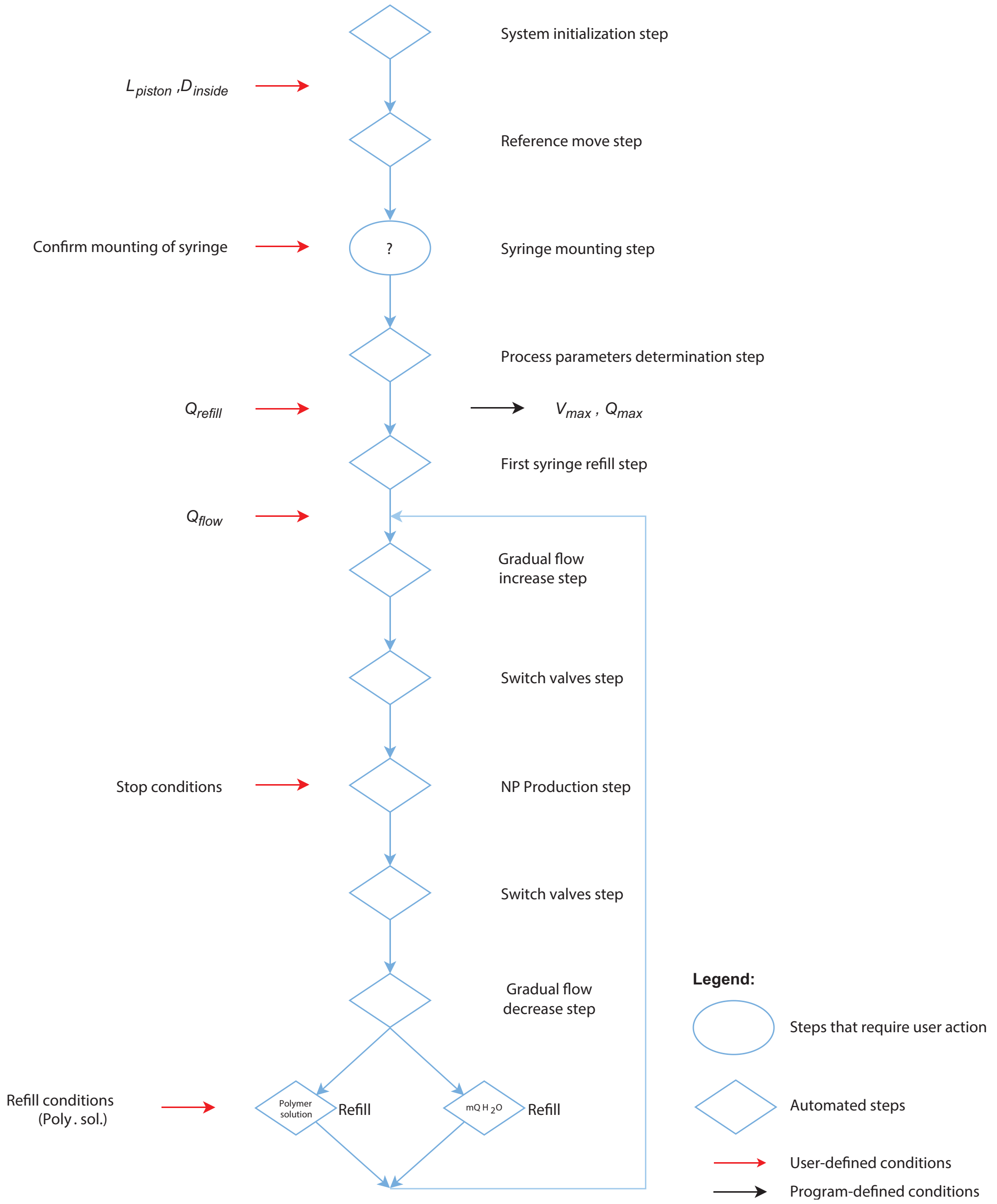

Figure S3. Block diagram illustrating the LabView program steps included in the NP production procedure.

Some of the production processes that were shown in this work required to change NP precursor solution during operation. To this aim, an automated washing procedure was implemented into the developed LabView program (Figure S4).

- The washing procedure started with the *empty polymer solution step* where the polymer solution syringe was emptied into the respective reservoir with a defined flow rate (Q_{Refill}).
- During the *exhange reservoirs step* the user had to switch the polymer solution reservoir with the desired washing solution and the NP solution container with a waste container. A confirmation is required by the user to continue the washing process.

- Subsequently, during the *refill all syringes step* and the *empty all syringes step* all syringes were sequentially refilled and emptied for a given number of cycles ($n_{washing}$). Between each step, the valves directed the flow to the desired reservoirs or to the CJM.
- At the end of the washing procedure all syringes were refilled (*refill all syringes step*) and the user as asked to confirm the start of the new NP production (*restart production step*).

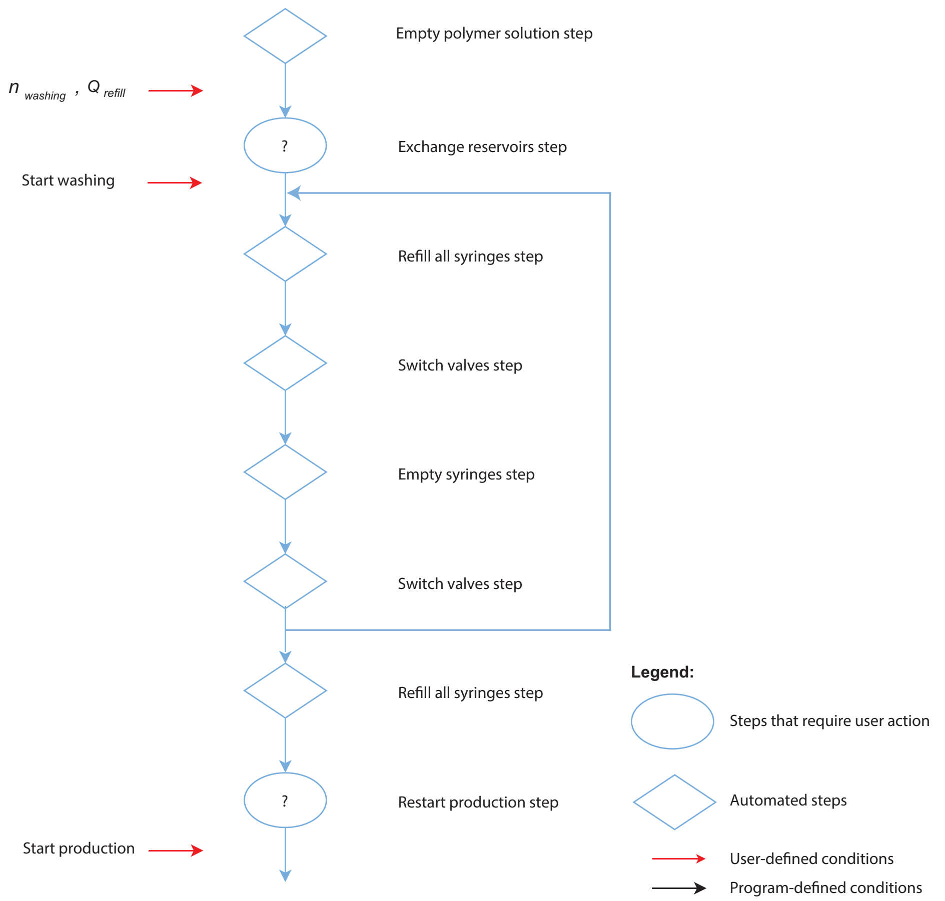

Figure S4. Block diagram illustrating the LabView program steps included in the washing procedure.

A rate of NP production was calculated to describe the productivity of the automated CJM. This parameter was calculated as follows:

$$\text{Prod. Rate} = \frac{C_{poly} \cdot V_{\text{polymer solution used}}}{t_{\text{NP synthesis}}} \tag{S1}$$

1.2 LabView script

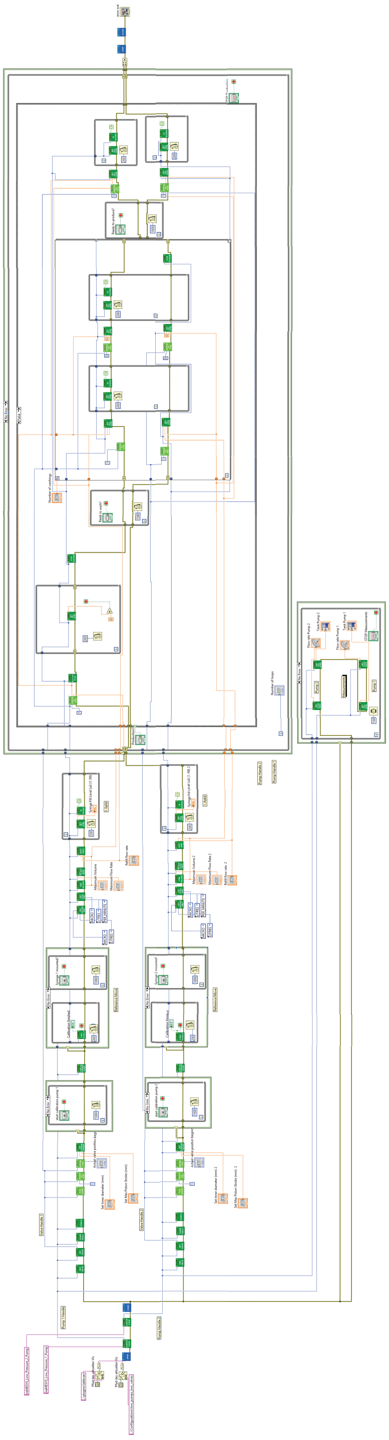

Figure S5. An illustration of the LabView script that was utlilized in this study.

1.3 Quantification of the NP Drug loading

A solution of 50 mg mL^{-1} of PEG_{5k}-*b*-PLA_{20k} and 0.5 mg mL^{-1} of OR was prepared for nanoprecipitation of OR-loaded NPs (tDL = 1%). That is, for the preparation of 1 mL NP precursor solution, 50 mg of PEG_{5k}-*b*-PLA_{20k} ($m_{\text{polymer in formulation}}$) and 0.5 mg of OR ($m_{\text{drug in formulation}}$) were dissolved in 1 mL of ACN. After nanoprecipitation, the OR content of the model drug-loaded NPs was quantified by utilizing the following procedure. After production, the NP suspensions were stored for 1 day at ambient temperature and for an additional day at 4 °C to promote crystal formation of non-encapsulated OR. The crystals formed were separated from the suspension via centrifugation at 7000 RCF for 15 min. 6 mL of crystal-free NP suspension was concentrated via ultrafiltration in centrifugal filters (Amicon Ultra-15, Ultracel membrane, MWCO ≈ 50 kDa; Millipore) at 4500 RCF for 10 min. 5 mL of dH_2O was added to the centrifugal filter in order to wash out free OR molecules in solution. Each water addition was follwed by centrifugation at 4500 RCF for 7 min. 50 μL of the concentrated NP suspension were diluted with 950 μL of ACN, sonicated for 10 min, and analyzed in a plate reader (Hidex Sense) to quantify OR at λ = 520 nm. The results from the spectrophotometer were compared to OR calibration curves and the total mass of drug in the filtered samples was determined ($m_{\text{drug after filtration}}$). The remaining NP suspension composed by both the block copolymer and the drug was lyophilized and its mass was quantified ($m_{\text{total formulation after filtration}}$).

1.4 Synthesis of PNP hydrogels

The NP suspensions were concentrated to 20 %w/w using centrifugal filters (Amicon Ultra-15, Ultracel membrane, MWCO ≈ 50 kDa; Millipore). To form 6.0 g of 2:15 %w/w PNP hydrogel, 0.120 g of HPMC were dissolved in 1.38 g of water over 12 h and added to a 10 mL plastic syringe. A syringe of equal volume was filled with the prepared 4.5 g of 20 %w/w NP solution. After eliminating residual bubbles via centrifugation, the two syringes were connected with a female-to-female luer lock adapter (Cellink) and mixed for 5 min (Figure S8).

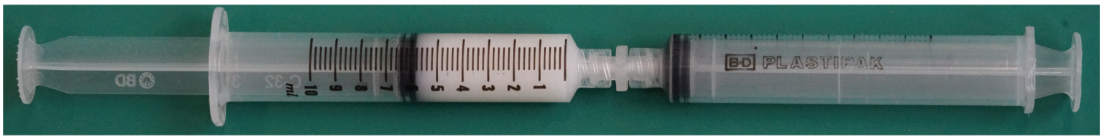

Figure S6. Syringe mixing

2 SUPPLEMENTARY RESULTS

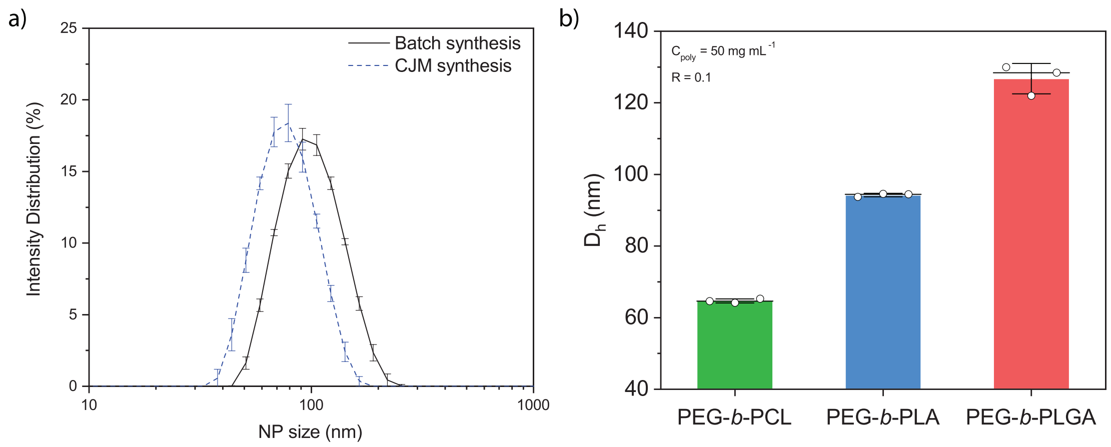

Figure S7. a) Size distribution of NPs produced from 50 mg mL^{-1} PEG-*b*-PLA solutions in ACN (R = 0.1). NPs produced by the automated CJM at Re = 1016 ($D_h = 78 \pm 1$ nm) were in a similar size range and quality to NPs produced via discontinuous batch nanoprecipitation ($D_h = 94 \pm 1$ nm). The data is plotted as mean $\pm$ S.D. over three independent measurements. b) Batch nanoprecipitation of 50 mg mL^{-1} block copolymer solutions (R = 0.1). PEG$_{5k}$-*b*-PCL$_{20k}$ and PEG$_{5k}$-*b*-PLA$_{20k}$ were dissolved in ACN, and PEG$_{5k}$-*b*-PLGA$_{20k}$ was dissolved in DMF.

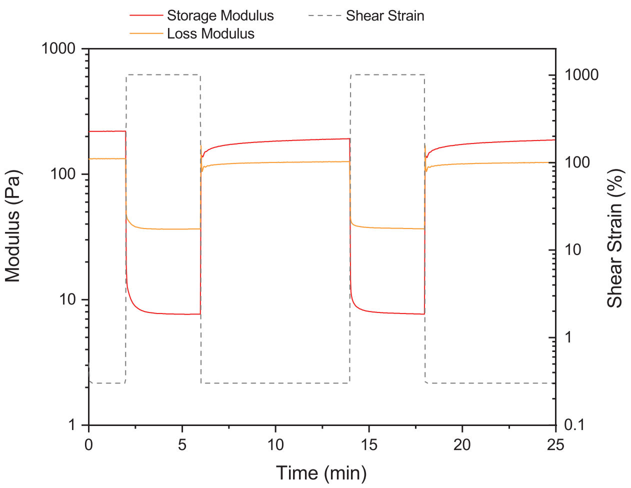

Figure S8. The self-healing behavior of the produced 0.6 g PNP hydrogels was characterized with oscillatory shear rheology. In the step strain test, the hydrogel network was broken with high strain intervals (γ = 1000%, ω = 10 rad s^{-1}). Subsequent low strain intervals allowed the material network to heal back (γ = 0.3%, ω = 10 rad s^{-1}).

Table S1. Continuous CJM production of PEG_{5k}-*b*-PLA_{20k}, n = 3 (Figure 2)

Subfig. [-]	Polymer [-]	Solvent [-]	Conc. [$mg\ mL^{-1}$]	*R* [-]	*Re* [-]	Q_{tot} [$mL\ min^{-1}$]	NP size [nm]	PDI [-]
a	PEG_{5k}-*b*-PLA_{20k}	ACN	10	0.005	1046	35	51 ± 1	0.08
b	PEG_{5k}-*b*-PLA_{20k}	ACN	10	0.005	1047	35	56 ± 1	0.06
b	PEG_{5k}-*b*-PLA_{20k}	ACN	10	0.005	538	18	79 ± 1	0.05
b	PEG_{5k}-*b*-PLA_{20k}	ACN	10	0.005	1047	35	56 ± 1	0.06
c	PEG_{5k}-*b*-PLA_{20k}	ACN	50	0.1	1016	35	75 ± 2	0.07
d	PEG_{5k}-*b*-PLA_{20k}	ACN	50	0.1	1016	35	74 ± 1	0.08
d	PEG_{5k}-*b*-PLA_{20k}	ACN	50	0.1	522	18	98 ± 1	0.08
d	PEG_{5k}-*b*-PLA_{20k}	ACN	50	0.1	1016	35	75 ± 1	0.08

Table S2. Batch nanoprecipitation of common block copolymers, n = 3 (Figure 3a)

Polymer [-]	Solvent [-]	Concentration [$mg\ mL^{-1}$]	*R* [-]	*Re* [-]	NP size [nm]	PDI [-]
PEG_{5k}-*b*-PCL_{20k}	ACN	10	0.005	-	55 ± 1	0.09
PEG_{5k}-*b*-PLA_{20k}	ACN	10	0.005	-	76 ± 1	0.07
PEG_{5k}-*b*-$PLGA_{20k}$	DMF	10	0.005	-	60 ± 5	0.09

Table S3. Continuous CJM production of common block copolymers, n = 3, over one cycle only (Figure 3c)

Polymer [-]	Solvent [-]	Concentration [$mg\ mL^{-1}$]	*R* [-]	*Re* [-]	NP size [nm]	PDI [-]
PEG_{5k}-*b*-PCL_{20k}	ACN	10	0.005	478	46 ± 1	0.04
PEG_{5k}-*b*-PLA_{20k}	ACN	10	0.005	1047	53 ± 1	0.06
PEG_{5k}-*b*-$PLGA_{20k}$	DMF	10	0.005	591	49 ± 1	0.07

Table S4. Continuous and stable CJM production of common block copolymers, n = 3, mean between all cycles (Figure 3e,f)

Subfig. [-]	Polymer [-]	Solvent [-]	Conc. [$mg\ mL^{-1}$]	*R* [-]	*Re* [-]	Q_{tot} [$mL\ min^{-1}$]	NP size [nm]	PDI [-]
e	PEG_{5k}-*b*-PCL_{20k}	ACN	10	0.005	478	16	46 ± 1	0.04
e	PEG_{5k}-*b*-PLA_{20k}	ACN	10	0.005	1046	35	53 ± 1	0.07
e	PEG_{5k}-*b*-$PLGA_{20k}$	DMF	10	0.005	591	20	49 ± 2	0.07
f	PEG_{5k}-*b*-PCL_{20k}	ACN	50	0.1	464	16	65 ± 1	0.03
f	PEG_{5k}-*b*-PLA_{20k}	ACN	50	0.1	1016	35	78 ± 1	0.08
f	PEG_{5k}-*b*-PLA_{20k}	ACN	50	0.1	522	18	97 ± 1	0.08
f	PEG_{5k}-*b*-$PLGA_{20k}$	DMF	50	0.1	833	35	103 ± 1	0.07

Table S5. Batch nanoprecipitation of common block copolymers, n = 3 (Figure S7b)

Polymer [-]	Solvent [-]	Concentration [$mg\ mL^{-1}$]	*R* [-]	*Re* [-]	NP size [nm]	PDI [-]
PEG_{5k}-*b*-PCL_{20k}	ACN	50	0.1	-	65 ± 1	0.05
PEG_{5k}-*b*-PLA_{20k}	ACN	50	0.1	-	94 ± 1	0.09
PEG_{5k}-*b*-$PLGA_{20k}$	DMF	50	0.1	-	127 ± 2	0.10
